# Heterologous Immunity of Virus-Specific T Cells Leading to Alloreactivity: Possible Implications for Solid Organ Transplantation

**DOI:** 10.3390/v13122359

**Published:** 2021-11-24

**Authors:** Gonca E. Karahan, Frans H. J. Claas, Sebastiaan Heidt

**Affiliations:** Department of Immunology, Leiden University Medical Center, 2333 ZA Leiden, The Netherlands; f.h.j.claas@lumc.nl (F.H.J.C.); s.heidt@lumc.nl (S.H.)

**Keywords:** viral infections, cross-reactivity, human leukocyte antigens (HLA), alloreactive memory, kidney transplantation

## Abstract

Exposure of the adaptive immune system to a pathogen can result in the activation and expansion of T cells capable of recognizing not only the specific antigen but also different unrelated antigens, a process which is commonly referred to as heterologous immunity. While such cross-reactivity is favourable in amplifying protective immune responses to pathogens, induction of T cell-mediated heterologous immune responses to allo-antigens in the setting of solid organ transplantation can potentially lead to allograft rejection. In this review, we provide an overview of murine and human studies investigating the incidence and functional properties of virus-specific memory T cells cross-reacting with allo-antigens and discuss their potential relevance in the context of solid organ transplantation.

## 1. Introduction

Solid organ transplantation is a life-saving treatment option for patients with end-stage organ failure. The beneficial effect of human leukocyte antigen (HLA) matching between patients and donors in transplantation outcomes has been shown in large scale studies [1,2]. However, due to the enormous polymorphism of the HLA antigens, it is almost impossible to find a completely matched donor organ for a particular patient, and therefore, patients regularly get transplanted with organs from partially or even fully HLA-mismatched donors. Immune responses induced by mismatched HLA can evoke both the cellular and humoral arm of the adaptive immune system. Consequently, transplant recipients rely on lifelong immunosuppressive treatments, which suppress the host immune response against the allograft in an antigen non-specific manner. Currently available immunosuppressive regimens mainly target T cells and have been successful in reducing acute cellular rejections and hence improving short-term allograft survival. Despite advances in immunosuppressive agents and transplant procedures, graft rejection, both acute and chronic, remain a significant barrier to long-term allograft survival [3,4].

**Figure 1:**
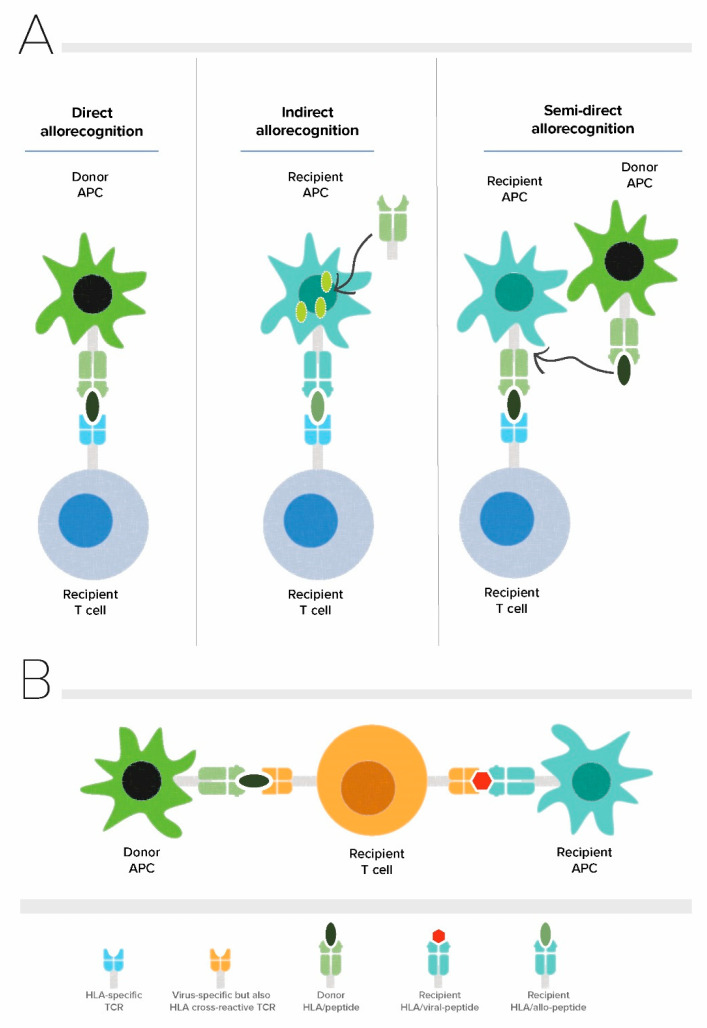
Allorecognition pathways and principle of heterologous immunity in alloreactive T cells. (**A**) Direct, indirect and semi-direct pathways of allorecognition (**B**) Allo-HLA cross-reactivity of virus-specific T cells. APC: Antigen presenting cell, TCR: T cell receptor.

Alloreactive T cells play a central role in mediating allograft rejection. The size and diversity of the alloreactive T cell repertoire is unique for every patient, and in addition to the degree of HLA matching, determines the strength of the immune response directed against the allograft [5,6]. In solid organ transplantation, alloreactive T cells recognize allo-antigens through direct, indirect, and semi-direct pathways (Figure 1A). The direct pathway of allorecognition is unique to allogeneic transplantation and involves CD8+ and CD4+ alloreactive T cells which are able to directly recognize intact HLA class I (HLA-A, -B, and -C) and HLA class II (HLA-DR, -DQ, and -DP) on donor cells, respectively [7]. Direct pathway alloreactive T cells are capable of conveying potent alloimmune responses, likely because of their high precursor frequency that arises due to lack of positive and negative selection to allogenic major histocompatibility antigens (allo-MHC) during thymic development [8,9]. Mature T cells are selected based on their intermediate affinity T cell receptor (TCR) recognition of self-peptide presented on self MHC in the thymus during their development whereas there is no selection based on their potential reactivity towards allogeneic MHC. In the context of allogeneic transplantation, T cells confuse peptide/allo-MHC complexes for foreign peptide/self MHC and deviate from the rule of MHC restriction [10,11,12]. Several molecular mechanisms enabling different docking modes of TCR have been proposed as the models underlying TCR cross-reactivity up to date and include TCR adaptation by induced fit, differential docking of TCR onto the peptide/MHC complex, structural degeneracy, molecular mimicry, and antigen-dependent tuning of peptide/MHC flexibility [9]. Among these, molecular mimicry which is a form of TCR degeneracy through which the TCR recognizes different peptide/MHC molecules sharing structural similarities, is the most likely mechanism for cross-reactivity of TCR with allo-antigens [12,13]. The indirect pathway of allorecognition is similar to conventional T cell responses mounted against common protein antigens, and involves alloreactive T cells of the recipient recognizing allogeneic HLA class I or class II-derived peptides presented on self HLA class II molecules. Finally, in the semi-direct pathway recipient, alloreactive T cells recognize intact allogenic HLA similar to the direct way of allorecognition but now on the surface of the self-antigen presenting cell (APC) that have acquired allogeneic HLA by various means such as cell to cell contact or exosomes. Altogether alloreactive T cells count up to 1–10% of peripheral T cells and consist of both naïve and memory T cells [14,15,16,17,18].

In transplantation, alloreactive T cells need to be kept in check by potent immunosuppressive drugs. Of the total T cell pool, memory T cells are potentially more difficult to suppress in comparison to their naïve counterparts due to their lower threshold for activation, independence of co-stimulatory signals, improved adhesion capacity, and stronger effector functions [19,20]. Alloreactive memory T cells can develop as a result of exposure to allo-antigens through blood transfusions, pregnancies, or previous transplantations. In healthy individuals, approximately 60% of the alloreactive T cell repertoire is composed of antigen-experienced memory T cell clones [21]. Several studies have shown an association of elevated pre-transplant frequencies of alloreactive memory T cells with an increased risk of acute rejection within the first year after kidney transplantation [22,23,24].

An individual’s T cell repertoire is not only shaped by thymic selection but also by exposure to environmental antigens and pathogens in the periphery. As T cells have not been positively nor negatively selected on allo-MHC during their development in the thymus, the mature circulating T cell repertoire of an individual has the potential to have TCRs with a wide range of affinities for allo-MHC, including TCRs with high affinity. The clonal distribution of this naïve repertoire is further shaped by adaptive immune responses to pathogens or vaccinations, generating memory T cells that are potentially cross-reactive with allo-MHC. Consequently, individuals without any prior exposure to allo-antigens can also harbour alloreactive memory as a result of a phenomenon known as heterologous immunity [11,12]. Heterologous immunity in the context of allogeneic transplantation refers to the cross-reactivity of virus-specific memory T cells with allo-antigens (Figure 1B) [13,14]. A classic and well-characterized example of this type of heterologous immunity has been demonstrated by Burrows and colleagues for CD8+ TCR bearing cells recognizing the Epstein-Barr virus (EBV) EBNA-3A antigen-derived FLRGRAYGL (FLR) peptide in the context of HLA-B*08:01, cross-reacting with allogeneic HLA-B*44:02 [13,25]. Considering that an individual will be exposed to an infinite number of viral infections throughout life, every individual’s T cell repertoire is expected to harbour a considerable number of virus-specific T cells with cross-reactive potential [26]. Indeed, cross-reactivity of virus-specific memory T cells with allo-antigens appears to be rather common and occurs in around 45% of virus-specific T cell clones and 80% virus-specific T cell lines generated from healthy individuals [27,28,29]. The cross-reactivity of the TCR with allo-antigens can be of clinical significance in the setting of solid organ transplantation because of the capacity of these memory T cells to directly recognize donor MHC/peptide complexes and their potential to cause allograft rejection in addition to hindering induction of transplant tolerance [30].

In this review, we present an overview of data from murine and human studies focusing on heterologous immunity occurring as a result of virus-specific TCR engagement with peptide/allo-MHC complexes and provide an insight into their possible clinical relevance to solid organ transplantation.

## 2. Relevance of Heterologous Immunity in Transplantation: Evidence from Murine Studies

None of the currently available immunosuppressive agents used in standard-of-care is antigen-specific and consequently, there is a continuous elevated risk of infection or cancer as a result of over-immunosuppression. This risk has to be balanced with the risk of allograft rejection as a result of under-immunosuppression. Therefore, tolerance induction, -long term acceptance of allografts in the absence of immunosuppressive treatment, while retaining protective immunity- has longtime been the ultimate goal in transplantation immunology research. Although successful attempts have been made in pathogen-free mice, only a few approaches found their way to clinical application in humans [31]. Among the murine studies, co-stimulation blockade with anti-CD40L antibody combined with donor-specific transfusion was shown to be successful in inducing tolerance in naïve mice receiving cardiac allografts, whereas mice with previously skin graft-primed memory T cells from the same strain as cardiac grafts were found to be resistant to the effects of co-stimulation blockade [20], showing that the presence of alloreactive memory T cells prevents the establishment of tolerance. In addition, Adams et al. demonstrated that tolerance induction strategies worked well in naïve mice receiving skin grafts whereas mice with a history of multiple infections were refractory to such tolerance induction regimens. These data revealed that prior encounter with pathogens has the potential to be a barrier for co-stimulation blockade-induced tolerance [30], likely due to the greater alloreactive potential of the memory T cell compartment. When the authors adoptively transferred memory T cells derived from previously skin-transplanted animals in varying doses to naïve mice followed by administration of tolerance-inducing regimens, they observed that tolerance induction was prevented in a dose-dependent manner, suggesting that a critical number of donor-specific memory T cells was necessary to resist tolerance and promote rejection in skin grafts [30]. Furthermore, Brehm et al. showed that lymphocytic choriomeningitis virus (LCMV)-specific CD8+ T cells isolated from mice with acute LCMV infection were capable of driving rejection of skin allografts when adoptively transferred to recipients with severe combined immunodeficiency, showing the impact of virus-induced alloreactive T cells on graft rejection [32].

## 3. Cross-Reactive Virus-Specific Memory T Cells: How Predictable Are They in Humans?

T cells convey responses against protein antigens upon binding to antigenic peptides presented on self-major histocompatibility complex (MHC) molecules through their TCR, a heterodimer composed of an alpha (α) and a beta (β) chain in the vast majority of T cells [33,34]. Complementarity determining region 3 (CDR3) domains of both TCR α and β chains contribute to the binding specificity of a TCR. [35,36]. Although TCRs are considered to be highly specific, some degree of cross-reactivity is an inherent feature of the TCR, which can be beneficial in combatting a wide array of pathogens [37,38,39]. However, when elicited against allo-antigens in the setting of solid organ transplantation, it has the potential to be detrimental to graft outcomes [40].

Cross-reactivity of HLA class I-restricted virus-specific CD8+ memory T cells with HLA-A and/or HLA-B antigens has been shown for EBV, CMV, Varicella Zoster virus (VZV), Herpes simplex virus-2 (HSV-2), and human immunodeficiency virus (HIV) [27,28,29,41,42,43]. In addition to these, our group identified EBV and CMV-specific CD8+ T cell clones cross-reacting with HLA-C, but not with non-classical HLA-E and HLA-G expressed on target cells [44]. Although textbook knowledge dictates that CD8+ T cells are restricted by HLA class I molecules and not by HLA class II, cross-reactivity of HLA-B and HLA-C restricted CMV-specific CD8+ T cells with HLA-DR has also been identified [27,45]. These data indicate that the cross-reactive potential of virus-specific T cells is huge. In contrast to the abundant number of cross-reactivities described for CD8+ memory T cells, only a few studies have described pathogen-specific CD4+ memory T cells cross-reacting with allogeneic HLA [27,46,47], likely due to the technical difficulties in their detection as well as their less pronounced role in direct allorecognition. However, based on the molecular mechanisms involved, a substantial cross-reactive potential of virus-specific CD4+ memory T cells is to be expected.

While the overall T cell repertoire is largely unique for a given individual, common TCRs between individuals can be identified. T cells with (almost) identical TCR usage among multiple unrelated individuals are called T cells with public TCRs and those that are unique to an individual are called private TCRs. While alloreactivity by private TCRs is currently impossible to predict and requires extensive screening of individual patients, information on public TCRs can aid in the prediction of alloreactivity. The classical model of heterologous immunity clearly illustrates the presence of both private and public TCRs for a single viral specificity. HLA-B8-restricted EBV/FLR-specific memory CD8+ T cells cross-react with HLA-B*4402, -B*44:05 and -B*55:01 but not with HLA-B*44:03 [48,49]. While the TCR involved in cross-reactivity with HLA-B*4402, -B*44:05 has been shown to be public (LC13) being expressed in multiple HLA-B8+ individuals with a history of EBV infection [50,51], cross-reactivity with HLA-B*55:01 has been shown to be mediated by a private TCR [27,51]. Individuals who harbor both HLA-B*08:01 and HLA-B*44:02 have different TCR rearrangements, which prevent autoimmunity owing to thymic education for self-tolerance on B*44:02, while retaining HLA-B8 FLR specificity [52,53]. In a recent study, Huisman et al. investigated the allo-HLA cross-reactivity of CMV, EBV, and Adenovirus-specific T cell populations against a panel of target cells covering 116 common HLA class I alleles. The authors found a higher frequency and diversity of HLA-cross-reactivity for HLA-B*08:01-restricted virus-specific T cells in comparison to HLA-A*01:01, HLA-A*02:01 or HLA-B*07:02 restricted ones, with more common cross-reactivity towards HLA-B alleles compared to HLA-A and HLA-C. [29]. These results suggested that cross-reactivity of virus-specific T cells was independent of the viral-specificity but was influenced by the self-HLA of the individuals in this study.

Whereas the majority of TCRs are expected to be unique to a given individual, making the prediction of a cross-reactive potential very difficult, a growing line of evidence suggests that public cross-reactivity of virus-specific memory T cells to allogeneic HLA may be more common than previously anticipated [28,54]. Noteworthy, by screening a small cohort of only 30 healthy individuals, and using a limited number of tetramers presenting dominant viral epitopes, three novel public TCRs were identified [28]. Thus, extending the knowledge of public TCRs with further studies may aid in the development of tools enabling better monitoring and prediction of patients at risk of generating harmful alloreactive memory T cell-derived responses. Such knowledge could be beneficial for deciding which immunosuppressive agents should be administered (see below), and in which patients, safe tapering of immunosuppression could be achieved [55].

## 4. Heterologous Immunity: Shaping the Alloreactive T Cell Repertoire in Humans

Generally, an infection with a single virus provokes a polyclonal immune response with the potential to generate a diverse allo-reactive T cell repertoire [56]. When virus-specific T cell clones isolated from healthy individuals were co-cultured with a panel of allogeneic target cells, it became clear that multiple CD8+ virus-specific T cell clones of the same individual proliferated in response to allo-stimulation [56]. One striking finding in this study was the cross-reactivity of CMV B35/IPS and CMV A2/NLV-specific CD8+ T cells from one individual with either HLA-B*B51:01, HLA-B*57:01, HLA*B58:01 and HLA-B39:01, HLA-B*50:01, respectively. Conversely, it was observed that stimulation with a single allogeneic HLA molecule in a one-way mixed lymphocyte reaction was also able to induce proliferation of T cells with multiple virus specificities in the same individual [49,56].

Transplant recipients are at high risk for infections because of their immunosuppressed state and are known to benefit from peri-transplant vaccinations, although efficacy could be lower in comparison to the healthy population due to suboptimal vaccine immunogenicity [57,58,59]. Hypothetically, not only naturally acquired viral infections, but also vaccinations have the potential to induce heterologous immunity [60]. In mixed lymphocyte cultures of solid organ transplant recipients who received a seasonal influenza vaccine, Danziger-Isokov et al. showed an increase in IFN-γ production after vaccination when compared to baseline pre-vaccination levels [57]. While the authors suggested that vaccination could have induced cellular alloreactivity, they did not elaborate whether such an alloreactive response was truly occurring as a result of vaccine antigen-specific T cells cross-reacting with allo-antigens. In our previous work, we have shown the emergence of VZV-specific T cells in a kidney transplant patient who converted seropositive after VZV vaccination. One of the TCRs recognizing VZV peptide presented in self HLA-A2 was shown to be capable of cross-reacting with HLA-B*55:02, suggesting a role for molecular mimicry as the underlying effect of vaccination on T cell alloreactivity [61]. While in this case, we could prove that successful live-attenuated VZV vaccination generated de novo HLA-specific alloreactive memory T cells, further studies are required to confirm whether induction of allo-reactive T cells is a common feature of vaccination, including the mRNA vaccines commonly used nowadays to protect against COVID-19 infections [62,63].

Whereas there is a large body of evidence showing that cross-reactivity of virus-specific memory T cells to allogeneic HLA antigens is very common [13,25,27,28,41,49,56,60,61,64,65], hardly any data exist for cross-reactivity between exposure to viral infections and allogeneic HLA by B cells or antibodies [60,66]. Recently, several groups investigated whether COVID-19 infections or SARS-CoV-2 vaccinations have an impact on HLA antibody profiles of transplant recipients [67,68]. While some studies did not find any impact on anti-HLA antibody profiles, others linked the emergence of donor-specific antibodies to heterologous immunity caused by COVID-19 infections or SARS-CoV-2 vaccinations. Noteworthy, such an effect of “heterologous immunity” is possibly attributable to the non-specific bystander activation of HLA-specific memory B cells by soluble factors released from virus-specific T cells rather than BCR cross-reactivity with multiple antigens [26,60,69]. In a systematic study, we investigated whether antibody cross-reactivity between viral antigens and HLA is common. By testing several virus-specific monoclonal antibodies (mAbs) against an array of HLA molecules and several HLA-specific mAbs against various viral antigens, we did not find any evidence for cross-reactivity between viral antigens and HLA at the level of monoclonal antibodies [46]. These data suggest that although exposure to pathogens can shape alloreactive T cell repertoire, no such influence exists for fully differentiated B cells, possibly due to the self-HLA/peptide complex-independent selection process of B cells during development. Whether potential cross-reactivity exists in immature B cell subsets remains to be defined.

## 5. Impact of Donor-HLA Cross-Reactive Virus-Specific T Cells on Allograft Rejection in Humans

In contrast to the clear evidence obtained from murine studies, which show that cross-reactive virus-specific T cells can hamper tolerance induction and promote allograft rejection, studies conducted in human transplant recipients did not provide any solid evidence for a worse clinical outcome deriving from heterologous immunity. Mifsud and colleagues were the first to report that cross-reactive HLA-B8-restricted EBV/FLR-specific memory CD8+ T cells can be detected in pre- and post-transplant peripheral blood samples from immunosuppressed lung transplant patients at a comparable frequency to that of healthy individuals [70], indicating that the cross-reactive T cell pool is not expanding upon allo-recognition under immunosuppression. In addition, while the cross-reactive EBV/FLR-specific T cells isolated from peripheral blood were cytotoxic and produced IFN-γ, cross-reactive T cells in the bronchoalveolar lavage possessed only cytotoxic capacity, implying a functional diversity between the cross-reactive virus-specific T cells in peripheral blood and the ones in allograft. Furthermore, no evident difference in clinical outcome within the first year after transplantation was found between EBV seropositive HLA-B8+ patients receiving HLA-B*44:02+ vs. HLA-B*44:03+ allografts in the absence of an active EBV infection.

Considering that the frequency of donor-specific memory T cells dictates their potential pathogenic effects [30], investigations at the time of active viral infection/viral re-activation may prove more information on the impact of cross-reactive T cells on allograft outcome. Nguyen et al. monitored the dynamics of cross-reactive CMV-specific T cells in lung transplant recipients before and after CMV re-activation [71]. The authors detected HLA-A*02:01-restricted CMV/NLV-specific CD8+ T cells cross-reacting with certain HLA-B27 alleles in a selected group of HLA-A2+ lung transplant recipients, and in one patient observed a significant increase in the frequency of cross-reactive CMV-specific T cells prior to detectable CMV re-activation [71]. The frequency of HLA-A*02:01-restricted CMV/NLV-specific CD8+ T cells decreased back to baseline levels after CMV viremia was cleared and remained stable in the presence of persistent alloantigen exposure. In addition, the transient increase in cross-reactive CMV-specific T cells of this patient was not associated with the poor clinical outcome, likely because the mismatched donor HLA was HLA-B*27:04 and not one of the cross-reactive HLA-B27 alleles (HLA-B*27:05, B*27:07 or B*27:09) [54].

The first ex-vivo analysis of cross-reactive EBV and CMV-specific T cells in kidney transplant patients was reported by Heutinck and colleagues [72]. The authors longitudinally screened the peripheral blood of patients who had CMV re-activation or primary EBV or CMV infection post-transplantation using viral peptide-HLA class I tetramer complexes upon co-culturing with donor or HLA-mismatched third-party cells. Donor cross-reactive T cells specific for a single viral epitope were detected only at a single time point before or after transplantation, whereas cross-reactivity to HLA-mismatched third-party donors were detected at several time points. As an explanation for the absence of donor cross-reactive T cells in peripheral blood following transplantation, the authors speculated on homing of the donor cross-reactive T cells to the allograft. Recently, Stranavova et al. showed the presence of donor HLA cross-reactive CMV/IE1 specific T cells in post-transplant kidney biopsies of patients with concomitant CMV infection and rejection, suggesting an effect of donor cross-reactive CMV-specific T cells on allograft outcome [73], and indicating that homing of cross-reactive T cells to the allograft is indeed occurring.

One reason for the general lack of association between the presence of cross-reactive virus-specific T cells and inferior graft survival in kidney and lung transplant patients could be tissue-specific peptide recognition by cross-reactive virus-specific T cells, suggesting that the particular peptide presented in allogeneic HLA recognized by virus-specific T cells in vitro might not be expressed in kidney or lung tissue [74]. Indeed, it has been shown that HLA-B*44:02+ proximal tubular epithelial cells and human umbilical vein endothelial cells were poor targets of HLA-B8-restricted EBV/EBNA3A-specific T cells due to lack of endogenous (EEYLQAFTY) peptide presentation on these epithelial and endothelial cells [48].

Avidity of the virus-specific T cell receptor for the peptide/MHC complex is another important factor that affects T cell activation and the ability of cross-reactive virus-specific memory T cells to kill allogeneic target cells. As TCR recognition of the peptide presented on allogeneic HLA is not restricted by the positive and negative thymic selection, a broader range of TCR avidity can occur. Indeed, in a previous study using cold target inhibition assay and high concentrations of viral peptide loading, we found a higher TCR avidity for the viral epitope in cross-reactive T cell clones in comparison to the allogeneic epitope. Interestingly, when suboptimal levels of the viral peptide were added, cellular lysis was higher for the allo-antigen suggesting a shift toward allo-peptide in TCR avidity [12,75]. In addition, in conditions of optimal viral peptide expression, CD8 blocking did not hamper anti-viral reactivity, whereas, at suboptimal viral-peptide concentrations, anti-viral reactivity was CD8 dependent. Although these results are mainly limited to the EBV B8/FLR model for which the cross-reacting allo-peptide (EETLQAFTY) is also known, they suggest that the TCR avidity relies on viral and allogeneic peptide expression. As TCR avidity can be higher or lower for the allo-epitope in comparison to its viral epitope, viral infections or re-activations can indirectly influence alloreactivity. Moreover, potent immunosuppression currently in use can be proposed as another reason for the lack of association between the presence of cross-reactive T cells and worse allograft outcome, a fact supported by the low rate of early rejections despite the high incidence of pre-existing allo-HLA cross-reactivity.

Current immunosuppressive treatments are remarkably efficient at preventing T cell-mediated rejections and result in excellent short-term patient and graft survival. Calcineurin inhibitors (CNI), namely cyclosporine and tacrolimus, are the cornerstones of the most commonly used immunosuppressive regimens in solid organ transplantation. While being very effective in suppressing both naïve and memory T cells, CNIs are nephrotoxic necessitating minimization or even conversion to another immunosuppressive agent in some patients [76,77,78]. In recent years, a high-affinity CTLA4-Ig variant (Belatacept) that blocks CD28-CD80/CD86 co-stimulation pathway in T cells has been put forward as a potential substitute for CNIs owing to its nephroprotection and specific targeting of co-stimulation molecules. However, its adoption in clinical practice has been limited partly due to a higher rate of acute cellular rejections in Belatacept-treated patients in comparison to Tacrolimus-based immunosuppression [77,79,80]. Considering that naïve T cells need CD28-mediated stimulation for their activation in contrast to lower activation thresholds of memory T cells that do not rely on co-stimulation, memory T cells may play a role in these rejections. Indeed, a study conducted in a non-human primate kidney transplant model has shown high pretransplant frequencies of CD28+ CD8+ memory T cells associated with rejection in Belatacept, but not in the Tacrolimus treated group [81]. Furthermore, recent studies found a higher incidence of CMV disease in patients treated with Belatacept-based maintenance immunosuppression [82,83], suggesting a potential role for co-stimulation independent rejection. This may have been caused by virus-specific memory T cells that were cross-reactive with the allogeneic donor HLA.

## 6. Future Directions and Concluding Remarks

While viral infections themselves already pose a major risk for immunocompromised transplant patients, the potential cross-reactivity of virus-specific memory T cells with donor allo-antigens can introduce additional complexity to the clinical management of transplant patients.

Virus-specific T cells have the potential to elicit detrimental immune responses against the allograft, as demonstrated by in vitro studies revealing their cross-reactivity with allogeneic HLA [27,28,29]. However, although studies in mice have shown that cross-reactive virus-specific memory T cells can cause allograft rejection [30,84], a significant impact in humans has not been shown so far in clinical studies [54,70,71,72,73,85]. Importantly, understanding the interaction of the cross-reactive TCR with the allo-peptide/MHC complex can help to better define the relevance of these virus-specific cross-reactive T cells in the setting of clinical transplantation. Accordingly, knowledge on the allo-peptide which is lacking for virtually all human virus-specific T cell cross-reactivities will enable TCR avidity and crystallography studies unraveling the structural mechanism of TCR cross-reactivities as well as providing knowledge on tissue specificity of the peptide.

Given that single viral infections are capable of generating cross-reactivity against multiple allogeneic-HLA antigens, one can expect that adult patients awaiting a transplant may have gathered a broad alloreactive potential as a result of their lifelong exposure to several viruses. Such knowledge is particularly important when seeking an alternative immunosuppressive treatment, such as the agents blocking co-stimulatory pathways, which can be successful at preventing priming of naïve donor-reactive T cells while leaving cross-reactive virus-specific memory T cells unaffected. Likewise, when calcineurin inhibitor-based immunosuppression, which is known to be very effective at hampering memory T cells, is minimized, virus-specific memory T cells may become a serious threat for transplantation outcomes. Therefore, future studies aiming at understanding the potential effect of immunosuppressive drugs on virus-specific cross-reactive T cells are warranted.
